# Analytical Solution for Predicting the Elastic Modulus of a Cement Slurry System with the Effect of Calcium Dissolution

**DOI:** 10.3390/ma17163927

**Published:** 2024-08-07

**Authors:** Fengyan Qi, Wenbing Song, Zhiwei Chen, Jian Zhang

**Affiliations:** 1 Jiyang College, Zhejiang A&F University, Shaoxing 311800, China; fengyanqi_zjyc@163.com; 2 School of Civil Engineering and Architecture, Zhejiang University, Hangzhou 310058, China; zhiweichen_zju@163.com

**Keywords:** cement slurry system, calcium dissolution, elasticity theory, composite sphere model, elastic modulus

## Abstract

The dissolution of calcium ions in concrete in a low-alkalinity environment is an important factor causing a significant increase in the porosity of internal concrete, leading to a deterioration in its mechanical properties and affecting the durability of the concrete structure. In order to improve the reliability of concrete durability design and significantly increase the service life of concrete structures located in soft water environments, it is crucial to establish an analytical method to predict the elastic modulus (*E_dc_*) of cement slurry systems suffering from calcium dissolution. Firstly, the hydrated cement particles are regarded as a three-phase composite sphere composed of unhydrated cement particles (UC), a high-density hydrated layer (H-HL), and a low-density hydrated layer (L-HL). By introducing the equivalent inclusion phase (EQ) composed of UC and H-HL, the three-phase composite sphere model can be simplified into an equivalent hydrated cement particle model composed of EQ and L-HL. Finally, the *E_dc_* of the two-phase composite sphere composed of the equivalent hydrated cement particles and the porosity of the dissolved cement slurry system are solved by using elasticity theory. The effectiveness of the developed analytical method is verified by comparing it with third-party numerical results. Based on this method, the effects of hydration degree, volume ratio of calcium hydroxide (CH) to hydrated calcium silicate (C-S-H), and volume ratio of inner C-S-H to outer C-S-H on the *E_dc_* of the dissolved cement slurry system are analyzed. The parameter analysis indicates that among the three influencing parameters, the hydration degree has the greatest effect on the *E_dc_* of the dissolved cement slurry system. This study provides an analytical method for predicting *E_dc_*, which can provide some references for the durability design of concrete after calcium dissolution.

## 1. Introduction

Concrete is an important civil engineering material with great advantages such as low cost, simple preparation, good moldability, high compressive strength, and a wide range of strength grades. These advantages have led to its extensive application in various harsh fields, such as marine engineering and hydraulic engineering. However, the durability and service life issues of concrete have become increasingly prominent, receiving widespread attention from the academic and engineering communities [1,2,3,4]. There are different reasons for the insufficient durability of concrete, and calcium dissolution is one of them. Each year, a great amount of manpower and resources are required to repair the durability failures caused by calcium dissolution [5,6,7,8,9]. Therefore, the durability issues of concrete caused by calcium dissolution have always been one of the important research topics.

In a normal water environment, the calcium dissolution characteristics of cement-based materials exhibit a very slow process. However, for infrastructure such as dams and nuclear power plants that have suffered from soft water environments for a long time, the continuous and rapid calcium dissolution of this kind of concrete structure cannot be ignored and must be given high attention to ensure their safety and durability. The elastic modulus of dissolved concrete has become a critical parameter for evaluating the mechanical performance of hydraulic concrete structures. Once this value is less than the prescribed value (usually 20 GPa), it is considered that the concrete structures cannot meet the bearing capacity requirements. C-S-H and CH are the reaction products of cement mixed with water, and their content plays an important role in the microstructure characteristics and mechanical properties of cement slurry systems [10,11,12,13,14,15]. In standard curing conditions, the chemical properties of C-S-H and CH remain stable. However, once cementitious materials are exposed to soft water or other salt-containing media for a long time, calcium ions in cement slurry systems continuously diffuse outward due to the concentration difference between the internal cement-based materials and external environments. This will lead to a continuous decrease in the calcium ion concentration in the inner cement slurry systems, triggering changes in the solid-liquid equilibrium of calcium ions between the pore solution and solid hydrates, resulting in the gradual dissolution of CH and C-S-H in the hydration products, which is known as calcium dissolution [16,17,18]. The calcium dissolution leads to an increase in the porosity of cementitious materials, resulting in the deterioration of their strength and mechanical properties [19,20,21,22]. To evaluate the mechanical properties of concrete after dissolution, especially for concrete structures such as dams and nuclear waste containers that are immersed in soft water or seawater for long periods of time, it is crucial to determine *E_dc_* with the effect of calcium dissolution. So far, researchers have extensively studied the impact of calcium dissolution on the mechanical degradation of cementitious materials using various techniques [23,24,25], including the mercury intrusion method, scanning electron microscopy testing, ultrasonic detection, X-ray diffraction analysis, EDTA titration, energy spectrum analysis, etc.

In order to evaluate the chemical change of cement-based materials after calcium dissolution, Carde et al. [26] revealed through experiments that the effect of CH decalcification on compressive strength was more obvious than porosity. After C-S-H decalcification, a more obvious effect on the compressive strength was found. Based on the experimental data, Carde proposed the prediction method to analyze the relationship between the loss rate of compressive strength and porosity. Based on the theoretical model of Carde, Mainguy et al. [27] established the balance relationship between solid calcium and free calcium dissolution in cement slurry systems and mortar, and the model for the evolution of porosity with the degree of calcium dissolution was proposed. In the numerical simulation, Patel et al. [28], based on the reaction transmission model using the Boltzmann method, established a three-dimensional microstructure model of the cement slurry system, considering the process of calcium dissolution. The simulation results revealed that the dissolution rate of calcium in the cement slurry system was proportional to the diffusion coefficient of calcium ions in the microstructure, and the high capillary porosity would accelerate the dissolution of calcium ions. In view of the multi-scale characteristics of concrete, Nie et al. [29] developed a multi-scale scheme to simulate the dissolution process of solid calcium in concrete. After applying this model, the feasibility and reliability can be proved by comparing the experimental data. By considering the coupling effect of chloride and calcium ion diffusion, Li et al. [30] established the diffusion scheme for quantitatively analyzing the calcium dissolution and chloride diffusion characteristics. The simulation results showed that the calcium dissolution led to an increase in concrete porosity, resulting in an increase in chloride diffusivity. Liu et al. [31] developed a numerical method for discussing calcium dissolution, ion diffusion, and ionic interaction in cement systems. The findings suggested that calcium dissolution predominantly accelerated chloride diffusion as a result of the increase in porosity and macropores. Additionally, the electrochemical coupling effect of different ions induced calcium dissolution at the beginning of cement hydration. Huo et al. [32] proposed a theoretical method to describe the characteristics of seepage, chemistry, and microstructure of concrete suffering from calcium dissolution and studied the effect of infiltration and dissolution on the porosity and mechanical properties of dams. In terms of the impact of calcium dissolution on mechanical behavior in cement slurry systems, Constantinides et al. [33] found two kinds of calcium silicate hydrate with different densities by using nanoindentation technology and pointed out that inner calcium silicate hydrate was less impacted by calcium dissolution compared to outer calcium silicate hydrate, but the volume ratio of the two would not change with the increase of calcium dissolution. Huang et al. [34] studied the mechanical properties of dissolved cement-based materials with various dissolution degrees through experiments. The results showed that calcium dissolution had a greater impact on the stress-strain relationship, and the compressive strength was linear with the dissolution degree. Chen et al. [35] studied the influence of carbonation on the mechanical properties of a dissolved cement slurry system and found that compressive strength was reduced by calcium dissolution at short-term carbonation curing, and the reduction trend would be accelerated with the increase in carbonation curing time. By immersing the specimens in ammonium chloride solution, Jiang et al. [36] studied the mechanical properties of high-belite paste and ordinary cement slurry systems after the accelerated calcium dissolution. The results showed that the macromechanical properties of two kinds of pastes were similar after dissolution, and the addition of fly ash had a significant effect on improving the mechanical properties of dissolved high-belite paste. Nguyen et al. [37,38] established a stress-strain model of dissolved concrete under cyclic loading by introducing an equivalent homogeneous medium and the continuous damage mechanics method, and the effectiveness of the model was proved by the experimental data. Based on the above literature review, existing calcium dissolution approaches for cement slurry systems mainly studied the evolution of dissolution depth, mass loss, porosity, and compressive strength with dissolution degree, but few theoretical methods were proposed to analyze the change characteristics between elastic modulus and dissolution degree. In practical engineering, the elastic modulus of the cement slurry system has been regarded as a crucial parameter for evaluating the mechanical properties of concrete structures. To facilitate engineers considering calcium dissolution in the durability design of practical concrete engineering, it seems critical to establish a simple and effective analytical approach for quantitatively evaluating the degradation law of the elastic modulus of the dissolved cement slurry system.

To address the aforementioned issue, this study proposes an analytical solution for calculating the *E_dc_* of a dissolved cement slurry system. Based on the three-phase composite sphere theory, the hydrated cement particle is considered to be a concentric sphere, which is composed of an unhydrated cement particle, an inner hydrated layer, and an outer hydrated layer. By repeatedly introducing the equivalent sphere model, a simple analytical method is established to predict the *E_dc_* of the dissolved cement slurry system. Finally, a quantitative analysis is conducted to assess the hydration degree and volume ratios of C-S-H to CH (*V*_C-S-H_/*V*_CH_) and inner C-S-H to outer C-S-H (*V*_C-S-Ha_/*V*_C-S-Hb_) on the *E_dc_* of the dissolved cement slurry system.

## 2. Methods

### 2.1. Modeling of Porosity Variation in a Dissolved Cement Slurry System

The hardened cement slurry system is primarily composed of three components: unhydrated cement, hydration products, and pores. Among them, the hydration products obtained from the reaction of cement particles with water mainly consist of C-S-H and CH. According to Powers’ model, the volume fraction of each component in the products is obtained as follows [39]:

(1) $$V_{\mathrm{hyd}}=\frac{0.68\alpha }{(w/c)+0.32}$$

(2) $$V_{\mathrm{gel}}=\frac{0.19\alpha }{(w/c)+0.32}$$

(3) $$V_{\mathrm{cap}}=\frac{(w/c)-0.36\alpha }{(w/c)+0.32}$$

(4) $$V_{\mathrm{uc}}=\frac{0.32(1-\alpha )}{(w/c)+0.32}$$

where *w*/*c* represents the water-cement ratio; *V*_hyd_, *V*_gel_, *V*_cap_, and *V*_uc_ represent the volume fractions of hydrated gel, gel pores, capillary pores, and unhydrated cement, respectively.

**Figure 1:**
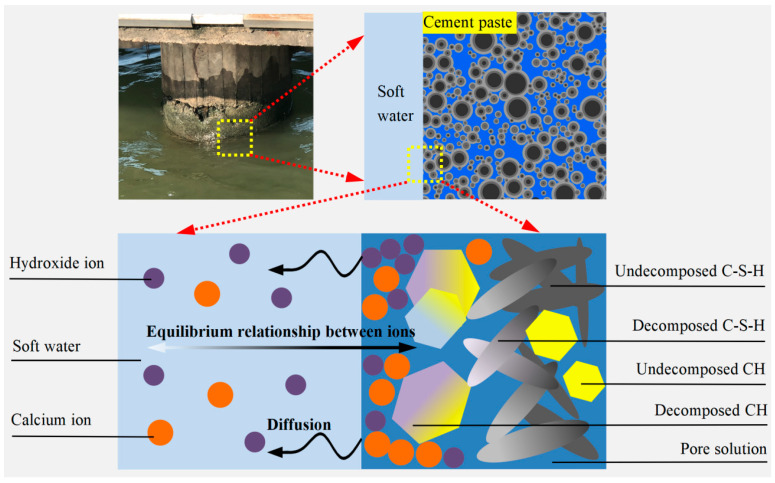
Calcium dissolution mechanism in a cement slurry system in a low-alkalinity environment.

According to Constantinides’ research [33], there are two different types of hydration gels formed from the hydrated cementitious materials. The main components of the hydration gels are C-S-H and CH. Hydrated calcium silicate can be divided into two categories: outer C-S-H_a_ and inner C-S-H_b_. The experimental results showed that the volume ratio of inner C-S-H to outer C-S-H increased from 0.33:0.67 to 0.30:0.70 before and after the calcium dissolution of the cement slurry system. According to this phenomenon, the volume ratio of the two kinds of C-S-H before the calcium dissolution can be approximately equal to the one after the dissolution. From a microscopic perspective, Figure 1. displays the dissolution mechanism of the calcium in the cement slurry system caused by soft water. From the figure, it can be seen that both calcium hydroxide and hydrated calcium silicate will decalcify, but the order of decalcification for the two is not consistent. As described by Berner [40], when the cement slurry system is eroded by soft water, the dissolution of calcium contains three main steps. The initial step is the decomposition of solid CH until all the solid CH is consumed. In the following step, the calcium in C-S-H is slowly dissolving. When the calcium concentration drops to about 2 mmol/L, the dissolution of calcium performs the last step, and partially dissolved C-S-H rapidly decomposes into silica gel. At this time, the calcium ion concentration in the pores is around 0–2 mmol/L. The solid-liquid equilibrium curve used is represented by the following equation [40]:

(5) $$us=\left\{\begin{array}{l} (-2u^{3}/x_{1}^{3}+3u^{2}/x_{1}^{2})[C_{\mathrm{CSH}}{\left(u/u_{\mathrm{satu}}\right)}^{1/3}],\;0\leq u\leq x_{1}\\ C_{\mathrm{CSH}}{\left(u/u_{\mathrm{satu}}\right)}^{1/3},\;x_{1}\leq u\leq x_{2}\\ C_{CSH}{\left(u/u_{\mathrm{satu}}\right)}^{1/3}+\frac{C_{\mathrm{CH}}}{{\left(u_{\mathrm{satu}}-x_{2}\right)}^{3}}{\left(u-x_{2}\right)}^{3},\;x_{2}\leq u \end{array}\right.$$

where *x*_1_ represents the calcium ion concentration in the pore solution when the C-S-H in the solid phase of calcium rapidly dissolves into silica gel; *x*_2_ represents the calcium ion concentration in the pore solution when the C-S-H starts to dissolve after the complete dissolution of CH in the solid phase of calcium; *u*_satu_ represents the saturation concentration of calcium ion in deionized water; and *C*_CH_ and *C*_CSH_ represent the initial concentrations of CH and C-S-H, respectively. In Equation (5), *x*_1_, *x*_2_, and *u*_satu_ can be fixed as 2, 17, and 20 mmol/L at room temperature [40].

Due to the dissolution of CH and C-S-H in the form of CH, the additional porosity caused by dissolution can be calculated by the following [41]:

(6) $$\varphi (x,t)=\varphi (x,0)+\frac{M_{\mathrm{CH}}}{\rho_{\mathrm{CH}}}[us(x,0)-us(x,t)]$$

According to the dissolution of different hydration products, the porosity of capillary pores and gel pores can be obtained by the following:

(7) $$\varphi_{\mathrm{cap}}(x,t)=\left\{\begin{array}{l} \varphi_{\mathrm{cap}}(x,0)+\frac{M_{\mathrm{CH}}}{\rho_{\mathrm{CH}}}[us(x,0)-us(x,t)],\;us(x,t)>C_{\mathrm{CSH}}^{0}\\ \varphi_{\mathrm{cap}}(x,0)+\frac{M_{\mathrm{CH}}}{\rho_{\mathrm{CH}}}C_{CH},\;us(x,t)\leq C_{\mathrm{CSH}}^{0} \end{array}\right.$$

(8) $$\varphi_{\mathrm{gel}}(x,t)=\left\{\begin{array}{l} \varphi_{\mathrm{gel}}(x,0),\;us(x,t)>C_{CSH}^{0}\\ \varphi_{\mathrm{gel}}(x,0)+\frac{M_{\mathrm{CH}}}{\rho_{\mathrm{CH}}}[us(x,0)-us(x,t)-C_{\mathrm{CH}}],\;us(x,t)\leq C_{\mathrm{CSH}}^{0} \end{array}\right.$$

where *φ*_cap_(*x*,0) and *φ*_gel_(*x*,0) represent the porosity of capillary and gel pores in the cement slurry system before dissolution, respectively; *φ*_cap_(*x*,*t*) and *φ*_gel_(*x*,*t*) represent the corresponding porosity in the cement slurry system at dissolution time *t* after dissolution, respectively; $C_{\mathrm{CSH}}^{0}$ is the initial concentration of C-S-H; *M*_CH_ is the molar mass of CH (74 g/mol); *ρ*_CH_ is the density of CH (2210 g/mol); *us*(*x*,0) is the initial concentration of soluble calcium in the solid phase before dissolution; *us*(*x*,*t*) is the concentration of soluble calcium in the solid phase at t after dissolution.

### 2.2. Modeling of Two-Phase Composites

**Figure 2:**
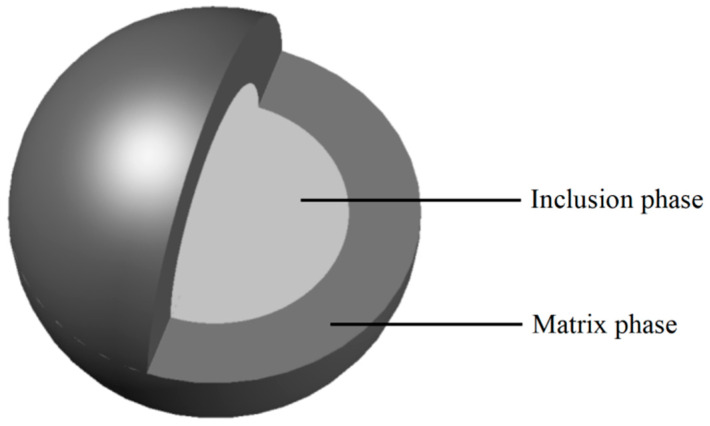
Two-phase composite sphere model.

The basic idea of constructing the elastic modulus of the two-phase composite sphere is shown below: Firstly, determine the basic mesoscopic parameters of composite materials, such as the shape and volume fraction of inclusions; secondly, establish a strengthening relationship between mesoscopic stress and macroscopic load; and finally, apply the mean field theory to replace heterogeneous elements with homogeneous material elements [5]. In this paper, the hydration product is regarded as the matrix phase, and the unhydrated cement particles are regarded as the inclusion phase. The two-phase composite sphere model can be seen in Figure 2. Since the cement hydration products are mainly composed of C-S-H and CH, and the CH is embedded around the C-S-H, it can be assumed that CH crystals are uniformly distributed in the matrix composed of C-S-H, and their shape is spherical [42]. Macroscopically, the homogenization method can be used to simulate composite materials in an equivalent medium with an effective elastic modulus. By using the elastic mechanics method, the effective bulk modulus of the two-phase composite sphere, which is composed of the inclusion phase and matrix phase shown in Figure 2, can be obtained as follows [43]:

(9) $$K_{e}=K_{m}+\frac{(K_{i}-K_{m})V_{i}}{1+[(1-V_{i})(K_{i}-K_{m})/(K_{m}+4G_{m}/3)]}$$

where *V_i_* represents the content of the inclusion phase, *K_i_* and *K_m_* represent the volume moduli of the inclusion phase and matrix phase, respectively, and *G_m_* is the shear modulus of the matrix phase.

The effective shear modulus *G_e_* of a composite sphere can be expressed as follows:

(10) $$G_{e}=G_{m}\frac{B\sqrt{B^{2}-4AC}}{2A}$$

where *A*, *B*, and *C* are functions related to the shear modulus and volume fraction of inclusions, defined as follows:

(11) $$\begin{array}{c} A=8(G_{i}/G_{m}-1)(4-5\mu_{m})\eta_{1}V_{i}^{10/3}-2[63(G_{i}/G_{m}-1)\eta_{2}+2\eta_{1}\eta_{3}]V_{i}^{7/3}\\ +252(G_{i}/G_{m}-1)\eta_{2}V_{i}^{5/3}-50(G_{i}/G_{m}-1)(7-12\mu_{m}+8\mu_{m}^{2})\eta_{2}V_{i}\\ +4(7-10\mu_{m})\eta_{2}\eta_{3} \end{array}$$

(12) $$\begin{array}{c} B=-4(G_{i}/G_{m}-1)(1-5\mu_{m})\eta_{1}V_{i}^{10/3}+4[63(G_{i}/G_{m}-1)\eta_{2}+2\eta_{1}\eta_{3}]V_{i}^{7/3}\\ -504(G_{i}/G_{m}-1)\eta_{2}V_{i}^{5/3}+150(G_{i}/G_{m}-1)(3-\mu_{m})\mu_{m}\eta_{2}V_{i}\\ +3(15\mu_{m}-7)\eta_{2}\eta_{3} \end{array}$$

(13) $$\begin{array}{c} C=4(G_{i}/G_{m}-1)(5\mu_{m}-7)\eta_{1}V_{i}^{10/3}-2[63(G_{i}/G_{m}-1)\eta_{2}+2\eta_{1}\eta_{3}]V_{i}^{7/3}\\ +252(G_{i}/G_{m}-1)\eta_{2}V_{i}^{5/3}+25(G_{i}/G_{m}-1)(\mu_{m}^{2}-7)\eta_{2}V_{i}\\ -(5\mu_{m}+7)\eta_{2}\eta_{3} \end{array}$$

*η*_1_, *η*_2_, and *η*_3_ are defined as follows:

(14) $$\eta_{1}=(G_{i}/G_{m}-1)(49-50\mu_{i}\mu_{m})+35(G_{i}/G_{m})(\mu_{i}-2\mu_{m})+35(2\mu_{i}-\mu_{m})$$

(15) $$\eta_{2}=5\mu_{i}(G_{i}/G_{m}-4)+7(G_{i}/G_{m}+4)$$

(16) $$\eta_{3}=(G_{i}/G_{m})(8-10\mu_{m})+(7-5\mu_{m})$$

where *μ_i_* and *μ_m_* represent the Poisson’s ratios of the inclusion phase and matrix phase, respectively.

When the bulk modulus and shear modulus of the two-phase composite sphere are obtained by solving Equations (9) and (10), the elastic modulus and Poisson’s ratio can be calculated as follows:

(17) $$E_{ie}=\frac{3}{1/Gie+1/3Kie}$$

(18) $$\mu_{ie}=\frac{Eie}{2Gie}-1$$

where *E_ie_*, *G_ie_*, and *K_ie_* represent the elastic modulus, shear modulus, and bulk modulus of the composite sphere and *μ_ie_* represents the Poisson’s ratio of the composite sphere.

### 2.3. Predicting the Elastic Modulus of the Dissolved Cement Slurry System

The analytical method is a computational approach that can effectively couple the multi-phase and multi-scale characteristics of cement-based materials and is often used for multi-scale prediction of the transport properties of cement-based materials. Due to the multi-phase and multi-scale microscopic composition and structural characteristics of the cement slurry system, this section will construct an analytical method to calculate *E_dc_* of the dissolved cement slurry system.

**Figure 3:**
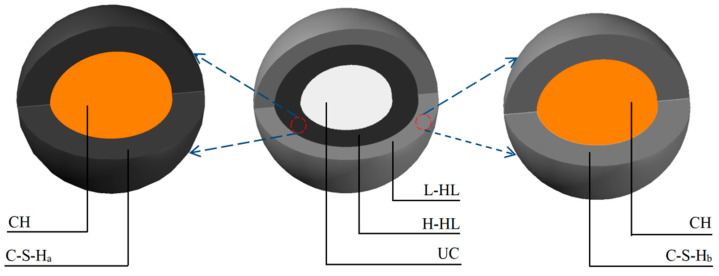
Composite sphere models with different hydration gels.

In the first step, the elastic modulus of the composite material composed of C-S-H and CH is determined. Of note, C-S-H contains two types: C-S-H_a_ and C-S-H_b_. The two-phase composite sphere model is illustrated in Figure 3. In this figure, the definitions of L-HL, H-HL, UC, CH, C-S-H_a_, and C-S-H_b_ can be found in the section above.

Based on former research, the volume ratio of CSH to CH in low-density hydration layer (L-HL) and high-density hydration layer (H-HL) can be approximated as 1.66:0.63 [3]. Therefore, the volume fraction of calcium hydroxide in hydration layers can be derived as follows:

(19) $$V_{\mathrm{CH}}=\frac{0.63}{0.63+1.66}=0.275$$

The volumetric modulus *K*, shear modulus *G*, and Poisson’s ratio *μ* of C-S-H_a_, C-S-H_b_, CH, and UC are shown in Table 1 [44]. Thus, according to Equations (9)–(18), the volume and shear modulus of L-HL and H-HL can be determined, and the corresponding results are also listed in Table 1.

**Figure 4:**
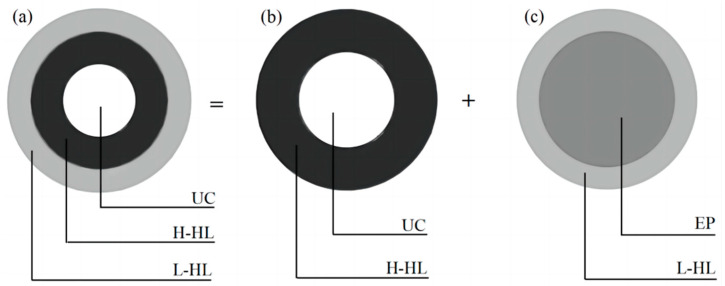
Three-phase composite sphere model is decomposed into two-phase composite spheres: (**a**) a three-phase composite sphere, (**b**) the first two-phase composite sphere, (**c**) the second two-phase composite sphere.

In the second step, the L-HL, H-HL, and unhydrated cement particles are simulated as a three-phase composite sphere, as shown in Figure 4a. Due to the difficulty in directly solving the elastic modulus of the three-phase composite material, the three-phase composite sphere can be decomposed into two two-phase composite spheres, as shown in Figure 4b,c. The first two-phase composite sphere consists of unhydrated cement and H-HL, where the unhydrated cement and H-HL are treated as the inclusion phase and matrix phase. The entire composite sphere can be regarded as an equivalent sphere. The second two-phase composite sphere consists of the equivalent sphere and L-HL, where the equivalent sphere and L-HL are treated as the inclusion phase and matrix phase. The volume modulus and shear modulus of the corresponding composite spheres can be obtained by using Equations (9)–(18). According to Constantinides’ research, *V*_C-S-Ha_/*V*_C-S-Hb_ is approximately 0.3:0.7, and *V*_C-S-Ha_ and *V*_C-S-Hb_ are to be free from the impact of the degree of calcium dissolution [33]. Therefore, the volume fraction of hydration layers can be calculated by the following:

(20) $$V_{L-HL}=\frac{0.476\alpha }{w/c+0.32}$$

(21) $$V_{H-HL}=\frac{0.204\alpha }{w/c+0.32}$$

where *V*_L-HL_ and *V*_H-HL_ represent the volume fractions of L-HL and H-HL, respectively.

Therefore, the volume fraction of unhydrated cement *V*_uc_ and equivalent sphere *V*_eq_ can be determined by the following:

(22) $$V_{\mathrm{uc}}=\frac{0.32(1-\alpha )}{0.32-0.116\alpha }$$

(23) $$V_{\mathrm{eq}}=\frac{0.32-0.116\alpha }{0.32+0.36\alpha }$$

In the third step, *φ*_cap_ after dissolution can be calculated by Equation (7). The pores are regarded as the inclusion phase, while the hydrated cement slurry system is considered the matrix phase. The elastic modulus of the dissolved cement slurry system is then solved by Equations (9)–(11). Due to the progress of calcium dissolution, the porosity of the cement slurry system increases gradually, and the elastic modulus of the cement slurry system decreases correspondingly. If the micropores are filled with water, the bulk modulus of the pore water is 2.2 GPa, the shear modulus is zero, and the Poisson’s ratio is 0.5. If there is no water in the micropores, both the bulk and shear moduli of the micropores are zero [42].

### 2.4. Experimental Verification and Discussion

**Figure 5:**
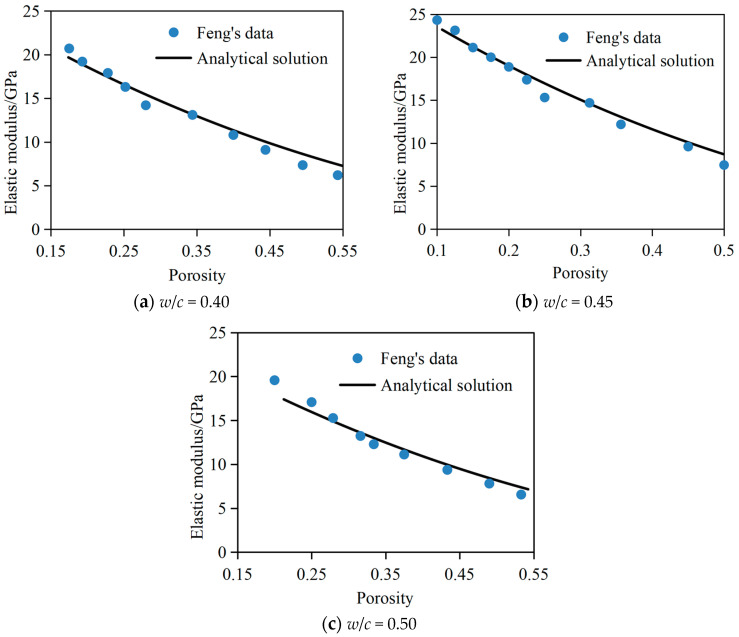
Comparison of analytical solution with third-party numerical results.

In order to verify the effectiveness of the developed analytical method, this section selects the results of Feng et al. [45] for comparison. In the simulation, the analytical solution of *E_dc_* of the dissolved cement slurry system was compared with the simulation results of Feng et al. when the water-cement ratio of the cement slurry system is 0.40, 0.45, and 0.50, as shown in Figure 5. It can be seen from the figure that the analytical solution in this paper is close to the results of Feng et al. [45], with correlation coefficients up to 0.96, 0.93, and 0.95, respectively, which verifies the effectiveness of the predicted method for the elastic modulus of the dissolved cement slurry system. It can also be observed from the figure that the elastic modulus of the cement slurry system decreases with the increase of porosity and water–cement ratio, and the main reason lies in the gain effect of porosity on the elastic modulus. Based on the predicted method for the *E_dc_* of the dissolved cement slurry system established in this paper, the key factors affecting the *E_dc_* of the cement slurry system will be analyzed quantitatively.

Considering the significant influences of three stages, namely initial calcium dissolution of CH, complete calcium dissolution of CH, and complete decalcification of C-S-H, on the *E_dc_* of the cement slurry system, Table 2 presents the evolution of the elastic modulus of the cement slurry system and the capillary porosity in these three stages. As can be seen from Table 2, within the fixed dissolution time, as *w*/*c* increases, porosity increases, while *E_dc_* decreases. When *w*/*c* varies from 0.4 to 0.5, *E_dc_* corresponding to initial calcium dissolution of CH, complete calcium dissolution of CH, and complete decalcification of C-S-H decreases by 23.9%, 20.6%, and 11.1%, respectively, which can be attributed to the fact that the initial capillary porosity increases with the increase of the water–cement ratio.

## 3. Results and Discussion

### 3.1. Effect of Hydration Degree on E_dc_

**Figure 6:**
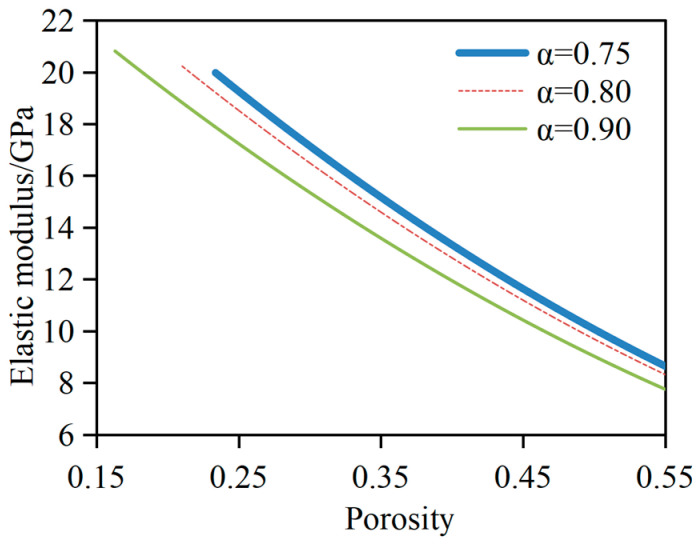
Effect of hydration degree on *E_dc_*.

In this parameter analysis, the water-to-cement ratio for the cement slurry system is fixed at 0.45. The degree of cement hydration is selected as 0.75, 0.80, and 0.90, respectively. The ratio of *V*_CH_ to *V*_C-S-H_ is taken as 0.275, and *V*_C-S-Ha_:*V*_C-S-Hb_ = 0.3:0.7. The change in *E_dc_* and porosity of the cement slurry system with different hydration degrees is shown in Figure 6. It can be observed that the higher the hydration degree, the smaller the initial porosity and the larger the elastic modulus of the cement slurry system before dissolution. For a given porosity, the elastic modulus of the cement slurry system decreases with increasing hydration degree. The simulated results indicate that when the porosity is 30%, 40%, and 50%, the elastic modulus of the cement slurry system at *α* = 0.75 is increased by 11.7%, 11.5%, and 11.2% compared to that of *α* = 0.90, respectively. The elastic modulus of the cement slurry system at *α* = 0.75 is increased by 4.0%, 3.9%, and 3.9% compared to that at *α* = 0.80, respectively. The elastic modulus of the cement slurry system at *α* = 0.80 is increased by 7.4%, 7.3%, and 7.2% compared to that at *α* = 0.90, respectively. Of note, under the same capillary porosity, the main reason for this phenomenon is that the lower the hydration degree, the higher the content of unhydrated cement, and the volume modulus and shear modulus of unhydrated cement are much larger than those of CH and C-S-H, resulting in an increase in the elastic modulus of the cement slurry system.

### 3.2. Influence of V_C-S-H_/V_CH_ on E_dc_

**Figure 7:**
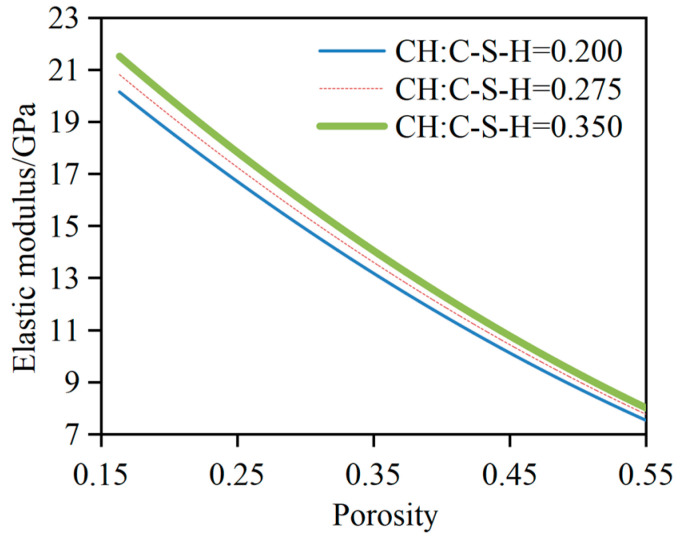
Influence of *V*_C-S-H_/*V*_CH_ on *E_dc_*.

In this parameter analysis, the w/c of the cement slurry system is set at 0.45, with a cement hydration degree of 0.90. *V*_C-S-H_/*V*_CH_ is taken at 0.20, 0.275, and 0.35, respectively. *V*_C-S-Ha_/*V*_C-S-Hb_ is fixed at 0.3:0.7. The relationship between the elastic modulus and porosity of the dissolved cement slurry system at different volume ratios of CH to C-S-H is shown in Figure 7. It can be observed from the figure that, for a given porosity, *E_dc_* increases with an increase in *V*_C-S-H_/*V*_CH_. Quantitative analysis results indicate that, when the porosity is 30%, 40%, and 50%, *E_dc_* at *V*_C-S-H_:*V*_CH_ = 0.35 increases by 6.6%, 6.4%, and 6.3%, respectively, compared to that at *V*_C-S-H_:*V*_CH_ = 0.20. The elastic modulus of the cement slurry system at *V*_C-S-H_:*V*_CH_ = 0.35 increases by 3.3%, 3.2%, and 3.2%, respectively, compared to that at *V*_C-S-H_:*V*_CH_ = 0.275. The main reason for this can be attributed to the fact that the volume modulus and shear modulus of CH are higher than those of C-S-H_a_ and C-S-H_b_, leading to an increase in the elastic modulus of the cement slurry system with an increase in the volume fraction of CH.

### 3.3. Influence of V_C-S-Ha_/V_C-S-Hb_ on E_dc_

**Figure 8:**
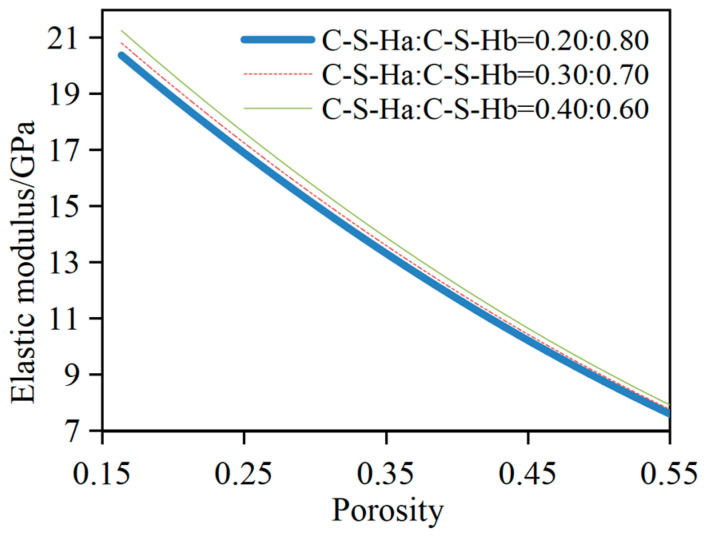
Influence of *V*_C-S-Ha_/*V*_C-S-Hb_ on *E_dc_*.

In this example, the cement slurry system with *w*/*c* = 0.45 and *α* = 0.90 is adopted. The *V*_C-S-H_:*V*_CH_ is fixed at 0.275. *V*_C-S-Ha_/*V*_C-S-Hb_ is set at 0.20:0.80, 0.30:0.70, and 0.40:0.60, respectively. The relationship between *E_dc_* and porosity at different C-S-H volume ratios is illustrated in Figure 8.

The figure shows that *E_dc_* increases with a rising *V*_C-S-Ha_/*V*_C-S-Hb_ for a specified porosity. Quantitative analysis has disclosed that for cement slurry systems with porosities of 30%, 40%, and 50%, the elastic modulus at *V*_C-S-Ha_/*V*_C-S-Hb_ = 0.40:0.60 exceeds those for the volume ratio of 0.20:0.80 by 4.3%, 4.2%, and 4.1%, respectively; for the volume ratio of 0.40:0.60, it is increased by 2.1%, 2.2%, and 2.0%, respectively, than those for the ratio of 0.30:0.70; and for the volume ratio of 0.30:0.70, it is increased by 2.1%, 2.0%, and 2.0% compared with the ratio of 0.20:0.80, respectively. The main reason can be ascribed to the following fact: At the equivalent degree of hydration, the initial porosity of the cement slurry system remains constant, and the hydrated cement particle consists of unhydrated layers, inner hydration layers, and outer hydration layers. Owing to the higher bulk and shear moduli of C-S-H_a_ relative to those of C-S-H_b_, *E_dc_* is observed to increase concomitantly with the increasing *V*_C-S-Ha_.

It should be gently reminded that the elastic modulus of the dissolved cement slurry system predicted by this analytical method only represents the elastic modulus at the linear elastic stage of the stress-strain curve. Actually, the stress-strain stage of a dissolved cement slurry system can be divided into a linear elastic stage, a non-linear elastic stage, a strain-hardening stage, and a failure stage. However, in traditional designs of concrete structures, engineers often pay the most attention to the linear elastic stage. In future research, by combining elastic-plastic theory and damage mechanics, the finite element method will be used to study the stress-strain relationship and peak strength of a dissolved cement slurry system.

## 4. Conclusions

This paper aims to comprehensively consider the complex multi-phase composition structure of the cement slurry system, namely the elastic modulus of unhydrated cement particles, calcium hydroxide, high-density hydrated calcium silicate, and low-density hydrated calcium silicate, before and after dissolution. Based on elastic theory and the composite sphere model, a step-by-step analytical method for solving the elastic model of dissolution cement slurry is derived, which can provide a new method for quantitatively evaluating the effect of important parameters on the elastic modulus of the dissolved cement slurry system. The main conclusions are as below:

(1) After the effectiveness of the analytical method is verified by Feng’s results, the effect of initial calcium dissolution of CH, complete calcium dissolution of CH, and complete decalcification of C-S-H on *E_dc_* and capillary porosity of the cement slurry system is analyzed. The calculation results show that within a given time, as the water-cement ratio and the dissolution degree increase, the capillary porosity increases while *E_dc_* decreases.
(2) The effect of degree of hydration, *V*_C-S-H_/*V*_CH_, and *V*_C-S-Ha_/*V*_C-S-Hb_ on *E_dc_* is analyzed. Calculation results show that when the porosity increases from 30% to 50%, *E_dc_* at *α* = 0.75 increases by about 11.6% compared to the one at *α* = 0.90. *E_dc_* at the volume ratio of CH to C-S-H of 0.350 increases by about 3.2% compared to the volume ratio of 0.275. *E_dc_* at *V*_C-S-Ha_/*V*_C-S-Hb_ = 0.40:0.60 increases by about 4.2% compared to the volume ratio of 0.20:0.80. The parameter analysis indicates that among the three influencing parameters, the hydration degree has the greatest effect on *E_dc_*.

## Figures and Tables

**Table 1:** Bulk moduli, shear moduli, and Poisson’s ratios of various constituents.

Hydration Product	*K*/GPa	*G*/GPa	*μ*
C-S-H_a_	18.88	11.85	0.240
C-S-H_b_	13.91	8.75	0.240
CH	33.39	14.50	0.310
UC	104.5	44.80	0.314
L-HL	17.36	10.03	0.258
H-HL	21.94	12.53	0.260

**Table 2:** *E_dc_* and capillary porosity at various dissolution stages.

Physical Variables at Different Stages		*w*/*c* = 0.40	*w*/*c* = 0.45	*w*/*c* = 0.50
Elastic modulus/GPa	Time when calcium ions begin to leach	24.3	20.8	18.5
Capillary porosity		0.089	0.139	0.186
Elastic modulus/GPa	Time when CH is depleted	15.5	13.6	12.3
Capillary porosity		0.287	0.322	0.356
Elastic modulus/GPa	Time when C-S-H is fully dissolved	4.5	4.3	4.0
Capillary porosity		0.582	0.599	0.617

## Data Availability

The original contributions presented in the study are included in the article, further inquiries can be directed to the corresponding authors.
